# Impact of Mycosis Fungoides/Sézary Syndrome on Patients’ and Cohabitants’ Quality of Life—A Cross-Sectional Study

**DOI:** 10.3390/jcm15062159

**Published:** 2026-03-12

**Authors:** Magdalena Łyko, Alina Jankowska-Konsur

**Affiliations:** University Centre of General Dermatology and Oncodermatology, Wroclaw Medical University, Borowska 213, 50-556 Wrocław, Poland

**Keywords:** quality of life, mycosis fungoides, Sézary syndrome, cohabitants, family

## Abstract

**Background/Objectives**: Mycosis fungoides (MF) and Sézary syndrome (SS) are chronic cutaneous T-cell lymphomas frequently associated with pruritus, psychological distress, and impaired quality of life (QoL). While the impact of MF/SS on patients’ quality of life is well recognized, data on the burden experienced by cohabitants remain limited. The aim of this study was to assess dermatology-specific quality of life in patients with MF/SS and their cohabitants and to explore its associations with pruritus severity, depressive symptoms, and disease stage. **Methods**: This cross-sectional study included 25 patient–cohabitant pairs (25 patients with MF/SS and their cohabitants living in the same household) recruited at a tertiary dermatology center. Patients completed the Dermatology Life Quality Index (DLQI), Beck Depression Inventory I (BDI-I), and pruritus intensity scales (Numeric Rating Scale and Visual Analogue Scale), whereas cohabitants completed the Family Dermatology Life Quality Index (FDLQI) to assess the family burden of the disease. Associations between quality-of-life measures, clinical characteristics, pruritus, and depressive symptoms were analyzed. **Results**: Patients reported moderate impairment in dermatology-specific quality of life (mean DLQI score of 9.3 ± 6.1), which was significantly greater in patients with advanced-stage disease (*p* = 0.022). Cohabitants also experienced moderate impairment in quality of life (mean FDLQI score of 8.0 ± 4.8), independent of disease stage. DLQI scores showed significant positive correlations with pruritus severity, depressive symptoms, and cohabitants’ FDLQI scores. Pruritus severity was a key determinant of impaired quality of life but did not differ significantly between disease stages. **Conclusions**: MF/SS are associated with a substantial multidimensional burden affecting both patients and their cohabitants. Quality-of-life impairment in family members correlates closely with patient-reported symptoms and well-being, supporting the concept of MF/SS as conditions affecting the patient–family unit. Incorporating caregiver perspectives and systematic symptom assessment may improve holistic management of MF/SS.

## 1. Introduction

Cutaneous T-cell lymphomas (CTCL) are a heterogeneous group of neoplasms primarily present in the skin. Mycosis fungoides (MF) is the most common subtype of CTCL that constitutes approximately 62% of all cases. It presents with erythematous patches and plaques that may progress to tumors or generalized erythroderma, typically accompanied by scaling. Sézary syndrome (SS) is a rare, aggressive leukemic variant of CTCL characterized by erythroderma, generalized lymphadenopathy, and the presence of malignant T cells in the peripheral blood. It constitutes 3% of CTCL cases [1]. MF/SS are frequently associated with subjective symptoms, including pruritus and a burning sensation of the skin. Pruritus is typically moderate to severe and significantly impairs quality of life [2]. Due to the chronic course of the disease and the need for long-term, often continuous treatment, patients repeatedly present to specialized centers and remain under ongoing medical supervision. Therapeutic strategies vary depending on the stage of the disease and include skin-directed therapies in early stages (such as topical corticosteroids, phototherapy, or local radiotherapy) as well as systemic treatments in advanced stages, including immunomodulatory agents, targeted therapies, biologic agents, and chemotherapy [3,4].

MF/SS impair patients’ quality of life due to chronic symptoms, visible skin changes, and the burden of long-term treatment. However, there is limited data on the disease burden on relatives of patients [5]. Recent reviews have highlighted needs in the assessment of health-related quality of life in patients with MF/SS, particularly regarding caregiver burden and the lack of comprehensive, disease-specific instruments [6]. Individuals living in the same household are often exposed to the daily challenges associated with the disease, including symptom management, treatment routines, and psychological distress. Unlike formal caregivers, cohabitants may not necessarily provide direct medical care but may still experience disease-related burden through shared daily life and emotional involvement. Our study addresses this gap by evaluating quality of life (QoL) in both patients and their cohabitants. The primary aim of this study was to assess dermatology-specific quality of life in patients with MF/SS and their cohabitants using DLQI and FDLQI. Secondary aims included evaluation of pruritus severity, depressive symptoms, and their associations with QoL outcomes. We hypothesized that greater symptom burden—particularly pruritus—and more advanced disease stage would be associated with worse quality of life in patients and greater QoL impairment among their cohabitants.

## 2. Materials and Methods

Patients diagnosed with MF/SS were recruited in the University Centre of General Dermatology and Oncodermatology, Wroclaw Medical University, Poland, from February 2022 to February 2025. A census sampling method was applied. All eligible patients were invited to participate; however, some declined participation. All patients included in the study were assessed during a disease exacerbation. Each participating patient was asked to identify one adult cohabitant living in the same household who was willing to participate in the study. Cohabitants were defined as partners or family members sharing the patient’s household. Inclusion criteria for patients were a diagnosis of MF/SS confirmed in histopathology and an age greater than 18 years. Inclusion criteria for family members were age greater than 18 years, having a close relationship with the patient, and living in the same household. Patients, partners, and relatives were excluded if they reported having any skin diseases, psychiatric conditions, or other significant illnesses that impaired their QoL, to avoid confusion about that impact.

Disease severity was determined according to the TNMB staging system for cutaneous T-cell lymphomas. Clinical staging was assessed based on clinical evaluation and available medical records at the time of study participation. Patients were subsequently categorized as having early-stage disease (IA–IIA) or advanced-stage disease (IIB–IV), and disease stage was used in the analyses, exploring associations with quality-of-life measures, pruritus severity, and depressive symptoms. Moreover, the skin disease severity was assessed using the modified Severity-Weighted Assessment Tool (mSWAT), which quantifies the extent and type of skin lesions in cutaneous T-cell lymphomas. Staging, classification of disease severity, and assessment of skin involvement were performed by M.Ł.

We used a specially designed questionnaire to collect participants’ demographic data. The impact of MF/SS on patients’ QoL was assessed using the 10-item Dermatology Life Quality Index (DLQI) [7]. The effect of MF/SS on cohabitants was measured with the validated Polish language version of Family Dermatology Life Quality Index (FDLQI) [8]. The severity of QoL impairment, as measured by the DLQI and FDLQI, was classified as follows: 0–1 points, no effect; 2–5 points, small effect; 6–10 points, moderate effect; 11–20 points, very large effect; and 21–30 points, extremely large effect.

Depressive symptoms were evaluated using the Beck Depression Inventory I (BDI-I), a 21-item self-report questionnaire validated for clinical and research use [9,10]. Each item is scored on a 4-point scale (0–3), yielding a total score of 0–63. The following cut-offs were applied: 0–9: minimal; 10–18: mild; 19–29: moderate; and 30–63: severe depressive symptoms. The questionnaire referred to the preceding week.

Pruritus intensity was assessed using both the Visual Analogue Scale (VAS) and the Numeric Rating Scale (NRS). Participants were asked to rate the severity of the most intense pruritus as well as the average pruritus experienced over the preceding 24 h. The VAS consisted of a 10 cm horizontal line anchored at 0 (“no itch”) and 10 (“worst imaginable itch”). The NRS required patients to select a number from 0 to 10, with 0 representing “no itch” and 10 representing “worst possible itch.” Cut-off scores for itch severity were defined as: 0, no itch; mild (<3), moderate (≥3–<7), severe (≥7–<9), and very severe (≥9) for both scales [11,12].

The DLQI, FDLQI, BDI, and demographic questionnaires were distributed to patients and their cohabitants during follow-up visits and could be completed either in the clinic or at home within 1–3 days, at the patient’s preference. The relationship of each cohabitant to the patient (parent, sibling, spouse, child, grandchild, or other) was recorded.

### 2.1. Statistical Analysis

Statistical analysis was performed using Statistica 13.0 software. Normality of continuous variables was assessed using the Shapiro–Wilk test. A Student’s *t*-test was used to compare the means of quantitative variables. When normality was not met, the Mann–Whitney U test was used instead. Qualitative variables were analyzed using the χ^2^ test, or Fisher’s exact test when at least one expected cell count was <5. Associations between continuous variables were examined with Pearson’s correlation coefficient, whereas Spearman’s rank correlation was applied for ordinal or non-normally distributed variables. A *p*-value of <0.05 was considered statistically significant. Given the relatively small sample size and the exploratory nature of this study, adjustments for multiple comparisons were not applied. Therefore, the results should be interpreted with caution.

### 2.2. Ethics

The study was conducted in accordance with the Declaration of Helsinki and was approved by the Institutional Review Board at Wroclaw Medical University (approval no. KB-925/2021). All patients provided informed consent, and the anonymity of all participants was strictly maintained.

## 3. Results

Of the 60 MF/SS patients and family members, six were excluded due to different places of residence. Two patients and two family members declined to participate and were unwilling to complete the questionnaire.

### 3.1. Clinical Characteristics of Patients and Cohabitants

In the final analysis, 50 participants were included: 25 MF/SS patients (23 MF and 2 SS) and 25 cohabitants. No rare clinicopathological variants were identified in the analyzed cohort. The mean patient age was 64.7 ± 14.2 (range: 32–84), with a median disease duration of 5 years. The mean age at onset was 57.4 ± 14.1. Most patients lived in urban areas (80%, *n* = 20) and were predominantly male (56%, *n* = 14). Based on TNMB staging, 18 patients (72%) had early-stage disease (IA–IIA), while 7 patients (28%) had advanced-stage disease (IIB–IIIA). The majority were diagnosed with stage IB (44%, *n* = 11). Educational level was evenly distributed between higher (40%, *n* = 10) and secondary education (40%, *n* = 10), while smaller proportions reported vocational (12%, *n* = 3) or primary education (8%, *n* = 2). Regarding treatment, the most common modality, besides topical corticosteroids used by all participants, was phototherapy or photochemotherapy (64%, *n* = 16), followed by methotrexate (16%, *n* = 4), and bexarotene (12%, *n* = 3). (Table 1) Among cohabitants, the majority were female (64%, *n* = 16) and most frequently spouses (68%, *n* = 17) (Table 2).

Disease severity measured with the mSWAT had a median of 13 points (range: 0–80). Pruritus intensity was generally moderate. The median average NRS score was 4.0 (Q1–Q3: 1.0–5.0), and the mean of the maximum NRS score was 4.6 ± 3.4. Similarly, the median for average VAS score was 3.9 (Q1–Q3: 1.0–4.8), while the maximum VAS score reached a mean of 4.7 ± 3.4. (Table 1).

The impact on dermatology-specific quality of life, measured with the DLQI, showed a mean score of 9.3 ± 6.1, corresponding to a moderate impairment. The FDLQI mean was 8.0 ± 4.8, also indicating a moderate impact on cohabitants’ quality of life. Depressive symptoms, assessed with the BDI, had a median score of 8.0 (Q1–Q3: 4.0–15.0).

### 3.2. Impact of MF/SS on QoL

**Figure 1:**
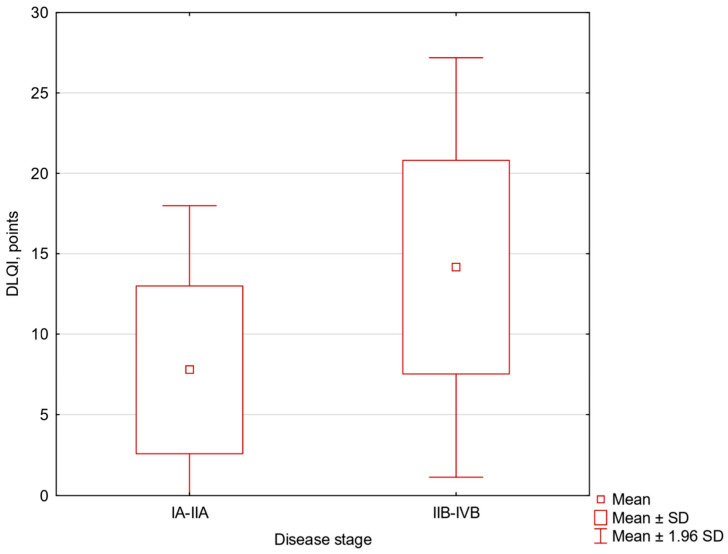
Dermatology Life Quality Index (DLQI) scores according to disease stage. The box-and-whisker plot shows DLQI scores in patients with early-stage (IA–IIA) and advanced-stage (IIB–IVB) cutaneous T-cell lymphoma.

Patients with advanced-stage disease reported significantly higher impairment in DLQI (t = −2.45, df = 23, *p* = 0.022, mean difference −6.377 (95% CI −11.752 to −1.002)) (Figure 1). No significant differences were observed between disease stage groups for pruritus intensity (VAS, NRS), FDLQI, or BDI scores.

**Figure 2:**
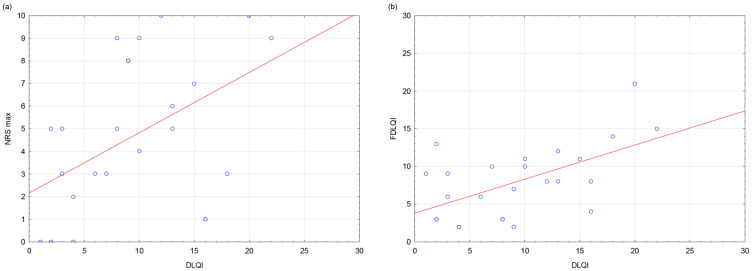
Correlations between patients’ quality of life, pruritus severity, and cohabitants’ quality of life. (**a**) Scatter plot showing the correlation between patients’ Dermatology Life Quality Index (DLQI) scores and maximum pruritus intensity assessed using the Numeric Rating Scale (NRS max). (**b**) Scatter plot showing the correlation between patients’ DLQI scores and Family Dermatology Life Quality Index (FDLQI) scores reported by cohabitants.

Significant positive correlations were observed between DLQI and measures of pruritus intensity. Higher DLQI scores were associated with greater itch severity assessed by both the NRS (average NRS: r = 0.44, *p* = 0.027; maximum NRS: r = 0.51, *p* = 0.017) and the VAS (average VAS: r = 0.41, *p* = 0.049; maximum VAS: r = 0.45, *p* = 0.023). (Figure 2a).

The positive correlation between DLQI and FDLQI was observed (r = 0.57, *p* = 0.003) (Figure 2b). Furthermore, DLQI correlated strongly with depressive symptoms measured by the BDI (r = 0.61, *p* = 0.001). Observed correlations showed moderate to strong effect sizes (r = 0.41–0.61).

Apart from its association with patients’ DLQI scores, no other significant correlations were observed between the total FDLQI score and clinical or symptom-related variables (Table 3).

### 3.3. DLQI Dimension Scores

Analysis of the DLQI subdomains revealed that the highest impairment was observed in the symptoms and feelings domain (items 1 + 2) with a median score of 2.0 (range 1–6). The daily activities domain (items 3 + 4) also showed considerable impact (median 2.0, range 0–6), followed by leisure activities (items 5 + 6) (median 1.0, range 0–5) and personal relationships (items 8 + 9) (median 1.0, range 0–6). In contrast, the work/school domain (item 7) and the treatment domain (item 10) were minimally affected, with median scores of 0.0 in both cases. Comparisons between patients with early-stage and advanced-stage disease were performed using the Mann–Whitney U test with continuity correction. Patients with advanced-stage MF/SS reported significantly greater impairment in the daily activities domain of the DLQI (items 3–4) compared with those with early-stage disease (U = 23.5, Z = −2.14, *p* = 0.032, r = 0.43). No statistically significant differences between disease stage groups were observed for the remaining DLQI subdomains (all *p* > 0.05).

### 3.4. FDLQI Dimension Scores

Descriptive analysis of FDLQI items demonstrated that the highest median scores were observed for increased household expenditure (item 10) and emotional distress (item 1). Time devoted to patient care (item 7) and physical well-being of family members (item 2) also showed moderate impairment. Other FDLQI domains showed lower median scores, indicating relatively limited impact on social life, leisure activities, and work or study (Table 4).

Analysis of FDLQI dimension scores by sex revealed a statistically significant difference only in dimension 6, with female cohabitants reporting higher impairment than male cohabitants (U = 39.0, Z = −2.33, *p* = 0.038, r = 0.47). When analyzed according to place of residence, a statistically significant difference was observed in dimension 9. Cohabitants living in rural areas reported greater impairment in this domain than those living in urban areas (U = 82.5, Z = 2.61, *p* = 0.024, r = 0.52).

## 4. Discussion

The present cross-sectional study demonstrates that MF/SS are associated with a moderate impairment of dermatology-specific quality of life not only in patients but also in their cohabitants. Significantly, patient-reported quality of life impairment was strongly associated with pruritus severity, depressive symptoms, and family members’ quality of life.

To date, only one study has specifically addressed the quality of life of family members of patients with MF. Dastgheib et al. [5] reported FDLQI outcomes in an Iranian cohort of MF patients and their relatives, demonstrating a moderate impairment of family QoL, with a mean FDLQI score of 8.44 ± 6.93. These findings are remarkably consistent with our results, in which family members also experienced a moderate burden, reflected by a mean FDLQI score of 8.0 ± 4.8. The close similarity in total FDLQI scores across two culturally distinct populations suggests that the family burden associated with MF may be a universal phenomenon, largely independent of sociocultural background. In both studies, emotional distress, caregiving demands, and financial burden emerged as key contributors to impaired family quality of life. Dastgheib et al. [5] identified extra expenditure, emotional strain, and caregiving responsibilities as the most affected FDLQI domains, which align closely with our domain-level findings, in which emotional burden, time devoted to care, and increased household expenditure were among the most affected areas. This concordance underscores the substantial and multidimensional impact of MF on the daily lives of patients’ relatives. However, some differences between the studies should be noted. In the Iranian cohort, family quality of life was significantly influenced by disease duration, head and neck involvement, and treatment with interferon, with greater impairment observed particularly during the early phase of the disease. In contrast, in our cohort, FDLQI scores were not significantly associated with disease stage or most sociodemographic variables. Still, they were strongly correlated with patient-reported quality of life and symptom burden, particularly pruritus severity. Our study extends these observations by incorporating both MF and Sézary syndrome patients and by directly examining the relationship between patient-reported quality of life (DLQI), family burden (FDLQI), pruritus intensity, and depressive symptoms.

The mean DLQI score in our cohort indicated moderate impairment of quality of life, consistent with previous studies reporting substantial dermatology-specific burden in patients with MF/SS [13,14,15]. In the study by Nourmohammadpour et al. [13], quality of life was evaluated in patients with early-stage mycosis fungoides, with particular emphasis on the influence of demographic and clinical subgroups on DLQI scores. While the overall DLQI impairment was comparable to that observed in our cohort, the domain-level analyses in that study were primarily conducted in relation to specific clinical and sociodemographic factors. For instance, greater impairment in the symptoms and feelings domain was observed in patients with both patch and plaque lesions compared with those with patch-only disease, and impairments in the personal relationships and treatment domains were associated with lower educational status [13]. Similar domain-level vulnerability has been reported by Graier et al. and in the PROCLIPI study, underscoring the central role of subjective symptoms and emotional well-being in MF-related burden [15,16]. In contrast to several previous reports, Nenonen et al. [14] found that patients with early-stage mycosis fungoides reported relatively little impairment in QoL, depressive symptoms, and pruritus. The authors attributed these findings to the characteristics of the Swedish healthcare system, including centralized care, extensive public funding, and the implementation of national treatment guidelines, which may have led to earlier diagnosis, better symptom control, and a higher proportion of patients with milder disease manifestations [14]. Importantly, Molloy et al. [16] identified female gender and alopecia as independent predictors of worse quality of life. However, data on the impact of gender on the QoL is inconsistent [13,14,17]. In our study, we did not observe significant gender-related differences in DLQI scores; this discrepancy may reflect differences in sample size, study design, and the quality-of-life instruments used.

Authors consistently highlight pruritus as a key determinant of quality-of-life impairment and emphasize that patients with more active or extensive disease represent a vulnerable subgroup requiring particular clinical attention [14,16,18]. Pruritus was associated with reduced quality of life in our study. Although itch intensity was generally moderate, higher pruritus severity assessed by both NRS and VAS was strongly associated with greater DLQI impairment. Roggo et al. [18] demonstrated that pruritus severity correlates more strongly with quality-of-life impairment than objective measures of skin involvement. Together, these observations support the concept that subjective symptom burden may outweigh disease extent in determining patient-reported outcomes in MF/SS. Notably, pruritus severity did not differ significantly between disease stages, suggesting that symptom burden is not solely dependent on the clinical extent of skin involvement. This finding supports the concept that even moderate itch may have a disproportionate impact on quality of life in MF/SS.

Depressive symptoms in our cohort were predominantly minimal; however, a subset of patients reported moderate to severe symptoms. The strong correlation between DLQI and BDI scores underscores the close interplay between impaired quality of life and psychological distress. Similar associations have been reported by Engin et al. [17] and by Graier et al. [15], despite differences in assessment tools and absolute depression scores. Notably, our study extends previous findings by demonstrating an association between pruritus severity and depressive symptoms and by highlighting the impact of patient-reported quality-of-life impairment on family members’ well-being. It should be noted that BDI was used as a screening tool and does not replace a formal psychiatric diagnosis.

### Limitations

This study has several limitations, including its cross-sectional design, relatively small sample size, and single-center setting, which may limit generalizability. In addition, participation of cohabitants was voluntary, which may have introduced selection bias, as individuals experiencing disease-related burden may have been more willing to participate. Furthermore, although major psychiatric disorders and psychiatric medication use were not reported in the studied cohort, other unmeasured psychosocial factors may have influenced depressive symptoms and quality-of-life outcomes. Additionally, causal relationships cannot be inferred. Nevertheless, the use of validated instruments and inclusion of both patients and cohabitants represent essential strengths. Despite these limitations, the observed associations showed moderate to strong effect sizes, supporting their clinical relevance.

## 5. Conclusions

This study provides a comprehensive assessment of the multidimensional burden imposed by mycosis fungoides and Sézary syndrome on both patients and their family members. Our findings demonstrate that MF/SS are associated with clinically meaningful impairment of dermatology-specific quality of life, even in predominantly early-stage disease, underscoring that limited clinical extent does not equate to limited patient burden.

Pruritus was associated with quality-of-life impairment, showing consistent associations with worse dermatologic QoL and increased depressive symptoms. Importantly, itch severity was not strictly dependent on disease stage, highlighting the need for systematic symptom assessment and management across the entire disease spectrum. The strong relationship between impaired quality of life and depressive symptoms further emphasizes the close interplay between physical symptoms and psychological well-being in MF/SS.

A key and novel contribution of this study is the demonstration that MF/SS substantially affects not only patients but also their cohabitants. The moderate impairment observed in family members’ quality of life, together with the strong correlation between patient- and family-reported QoL measures, provides quantitative evidence that MF/SS should be viewed as a condition affecting the patient–family unit rather than the patient alone. These findings support the inclusion of caregivers and family members in clinical assessment and supportive care planning.

From a clinical perspective, our results highlight the importance of a holistic, patient-centered approach to MF/SS management that extends beyond objective disease severity. Routine incorporation of quality-of-life instruments, pruritus scales, and screening for psychological distress may facilitate earlier identification of unmet needs and support more individualized care. In addition, assessment of family-related quality of life may help clinicians better capture the broader psychosocial impact of the disease and guide supportive interventions.

## Figures and Tables

**Table 1:** **Demographic characteristics of patients with MF/SS.** Data are presented as mean ± standard deviation (SD) for variables with normal distribution and as median (Q1–Q3) for variables with non-normal distribution. Categorical variables are presented as number (percentage).

Characteristics	Values
Gender, *n* (%)	
Female	11 (44)
Male	14 (56)
Age (years), mean ± SD	64.8 ± 14.2
Age at onset (years), mean ± SD	57.6 ± 14.1
Disease duration (years), median (Q1–Q3)	5.0 (3.0–9.0)
mSWAT, points; median (range)	13.0 (0–80)
Stage of disease, *n* (%)	
IA	4 (20)
IB	11 (44)
IIA	3 (12)
IIB	5 (20)
IIIA	2 (8)
Education, *n* (%)	
Academic degree	10 (40)
Secondary education	10 (40)
Primary education	2 (8)
Vocational education	3 (12)
Marital status, *n* (%)	
Single	8
Married	17
Residence, *n* (%)	
Urban	20 (80)
Rural	5 (20)
Therapy, *n* (%)	
Topical glucocorticosteroids	25 (100)
Phototherapy/Photochemotherapy	16 (64)
Methotrexate	4 (20)
Bexarotene	3 (12)
Itch severity	
NRS average, median (Q1–Q3)	4.0 (1.0–5.0)
NRS max, mean ± SD	4.6 ± 3.4
VAS average, median (Q1–Q3)	3.9 (1.0–4.8)
VAS max, mean ± SD	4.7 ± 3.4
BDI, points, median (Q1–Q3)	8.0 (4.0–15.0)
DLQI, points, mean ± SD	9.3 ± 6.1

SD—standard deviation; *n*—number; mSWAT—The modified Severity-Weighted Assessment Tool; NRS—numeric rating scale; VAS—visual analogue scale; BDI—Beck Depression Inventory; DLQI—dermatology life quality index.

**Table 2:** **Demographic characteristics of cohabitants.** Data are presented as mean ± standard deviation (SD) for variables with normal distribution and as median (Q1–Q3) for variables with non-normal distribution. Categorical variables are presented as a number (percentage).

Cohabitants’ Gender	
Male, *n* (%)	9 (36)
Female, *n* (%)	16 (64)
Cohabitants’ age (years), mean ± SD	58.9 ± 15.2
Cohabitants’ relation to patient, *n* (%)	
Spouse	17 (68)
Sibling	1 (4)
Parent	1 (4)
Child	5 (20)
Grandchild	1 (4)

SD—standard deviation; *n*—number.

**Table 3:** Correlation between patients’ clinical features and FDLQI scores in cohabitants.

Characteristics	r	*p*-Value
Patient’s age	0.232	0.26
Patient’s age at onset	0.132	0.53
Disease duration	−0.122	0.56
mSWAT	0.252	0.22
Itch severity		
NRS average	0.16	0.44
NRS max	0.23	0.25
VAS average	0.15	0.46
VAS max	0.19	0.36
BDI	0.38	0.06
DLQI	0.57	**0.003**

mSWAT—The modified Severity-Weighted Assessment Tool; NRS—numeric rating scale; VAS—visual analogue scale; BDI—Beck depression inventory; DLQI—dermatology life quality index. Statistically significant correlation are highlighted in bold.

**Table 4:** Impact of MF/SS on the individual items of the FDLQI.

FDLQI Items	Cohabitants Total	
	Mean ± SD	Median (IQR)
Over the last month, how much emotional distress have you experienced due to your relative’s/partner’s skin disease (e.g., worry, depression, embarrassment, frustration)?	1.24 ± 0.83	1 (1)
Over the last month, how much has your relative’s/partner’s skin disease affected your physical well-being (e.g., tiredness, exhaustion, sleep or rest disturbance)?	1.0 ± 0.65	1 (0)
Over the last month, how much has your relative’s/partner’s skin disease affected your personal relationships with him/her or with other people?	0.64 ± 0.86	0 (1)
Over the last month, how much difficulty have you had with other people’s reactions to your relative’s/partner’s skin disease (e.g., bullying, staring, or the need to explain the condition to others)?	0.4 ± 0.58	0 (1)
Over the last month, how much has your relative’s/partner’s skin disease affected your social life (e.g., going out, visiting or inviting people, attending social gatherings)?	0.52 ± 0.6	0 (1)
Over the last month, how much has your relative’s/partner’s skin disease affected your recreation or leisure activities (e.g., holidays, hobbies, gym, sports, swimming, watching television)?	0.6 ± 0.6	0 (1)
Over the last month, how much time have you spent looking after your relative/partner (e.g., applying creams, administering medications, or caring for their skin)?	1.04 ± 0.7	1 (1)
Over the last month, how much additional housework have you had to do because of your relative’s/partner’s skin disease (e.g., cleaning, vacuuming, washing, cooking)?	0.8 ± 0.64	1 (1)
Over the last month, how much has your relative’s/partner’s skin disease affected your work or studies (e.g., taking time off, reduced working hours, or difficulties at work)?	0.44 ± 0.51	0 (1)
Over the last month, how much has your relative’s/partner’s skin disease increased your routine household expenditure (e.g., travel costs, special products, creams, cosmetics)?	1.28 ± 0.73	1 (1)

SD—standard deviation; IQR—interquartile range.

## Data Availability

The data that support the findings of this study are available from the corresponding author upon reasonable request.

## Abbreviations

The following abbreviations are used in this manuscript:

MF	Mycosis fungoides
SS	Sézary syndrome
DLQI	Dermatology Life Quality Index
FDLQI	Family Dermatology Life Quality Index
BDI	Beck Depression Inventory
QoL	Quality of Life
VAS	Visual Analogue Scale
NRS	Numeric Rating Scale
